# From Open to Robot-Assisted Pancreatoduodenectomy: What RCTs Really Show

**DOI:** 10.3390/jcm15031225

**Published:** 2026-02-04

**Authors:** Alice Cattelani, Roberto M. Montorsi, Alessio Marchetti, Lucia Landi, Federico Gronchi, Matteo De Pastena, Luca Landoni, Alessandro Esposito, Salvatore Paiella, Giuseppe Malleo, Roberto Salvia

**Affiliations:** 1 Department of General and Pancreatic Surgery, The Pancreas Institute, University of Verona Hospital Trust, 37134 Verona, Italy; alice.cattelani@outlook.it (A.C.); montorsi.imd@gmail.com (R.M.M.); alemarche055@gmail.com (A.M.); lucialandi1999@gmail.com (L.L.); gronchifederico@gmail.com (F.G.); matteo.depastena@aovr.veneto.it (M.D.P.); luca.landoni@aovr.veneto.it (L.L.); alessandro.esposito@aovr.veneto.it (A.E.); salvatore.paiella@univr.it (S.P.); giuseppe.malleo@univr.it (G.M.); 2 Department of Surgery, Amsterdam UMC, University of Amsterdam, 1105 AZ Amsterdam, The Netherlands; 3 Cancer Center Amsterdam, 1081 HV Amsterdam, The Netherlands; 4 Pancreatic Surgery Unit, Department of Surgery, Dentistry, Paediatrics and Gynecology, University of Verona, 37134 Verona, Italy; 5 Pancreatic Surgery Unit, Department of Engineering for Innovation Medicine (DIMI), University of Verona, 37134 Verona, Italy

**Keywords:** pancreatic surgery, robotic surgery, laparoscopic surgery

## Abstract

**Introduction**: Minimally invasive pancreatoduodenectomy (MIPD), including laparoscopic (LPD) and robotic approaches (RPD), has gained increasing attention as an alternative to open pancreatoduodenectomy (OPD). Despite rapid technological progress, concerns persist regarding safety, reproducibility, and oncological adequacy. The publication of randomized controlled trials (RCTs) provides essential high-level evidence to reassess the true benefits and limitations of MIPD. **Methods:** This narrative review synthesizes all available RCTs comparing LPD and RPD with OPD. Major domains evaluated include mortality, major morbidity, intraoperative parameters, postoperative recovery, oncological outcomes, conversion, costs, and the influence of surgeon experience and institutional volume. The objective is to contextualize RCT findings rather than perform a quantitative meta-analysis. **Discussion**: Across studies, LPD demonstrates comparable mortality and complication rates to OPD in high-volume centers, with consistent reductions intraoperative blood loss (IBL) and shorter recovery or length of stay (LOS). RPD shows more heterogeneous results: one large trial reported improved postoperative recovery, whereas the EUROPA trial identified higher rates of pancreatic fistula (POPF) and delayed gastric emptying (DGE) alongside significantly increased costs. Both LPD and RPD achieve oncological outcomes equivalent to OPD, and 3-year survival data confirm the long-term non-inferiority of LPD. However, operative time remains longer for all minimally invasive approaches, and conversion persists as a marker of technical difficulty and incomplete learning curve. **Conclusions**: Current RCT evidence indicates that MIPD is safe, feasible, and oncologically sound only when performed by surgeons who have surpassed the demanding learning curve within specialized, high-volume centers. The benefits, mainly reduced IBL and faster recovery, must be weighed against longer operative times, conversion risks, and substantially higher costs for RPD. MIPD should therefore be considered an advanced option rather than a universal standard, and its broader implementation requires structured training pathways, appropriate patient selection, and institutional readiness.

## 1. Introduction

Pancreatoduodenectomy (PD), historically known as the Whipple procedure [1], stands as one of the most technically demanding operations in the surgical field. For decades, open pancreatoduodenectomy (OPD) has been the unchallenged gold standard for the management of benign and malignant pathologies of the pancreatic head and periampullary region. Despite significant advancements in surgical technique, perioperative care, and centralization of services, OPD continues to be associated with considerable morbidity, with postoperative complication rates historically reported as high as 30–50% [2], including potentially life-threatening events like postoperative pancreatic fistula (POPF) [3], delayed gastric emptying (DGE) [4], and postpancreatectomy hemorrhage (PPH) [5].

The late 20th and early 21st centuries witnessed a paradigm shift in surgery with the advent and widespread adoption of minimally invasive (MI) techniques [6,7]. Laparoscopic surgery, championed for procedures like cholecystectomy and appendectomy, promised and delivered reduced surgical trauma, less postoperative pain, shorter length of hospital stays (LOS), and faster return to normal activity [8,9]. This revolution naturally extended its reach to more complex oncological procedures, including colorectal and gastric resections [10]. The first laparoscopic pancreatoduodenectomy (LPD) was reported in 1994 by Gagner and Pomp, yet for many years it remained a curiosity, performed in a handful of pioneering centers [11]. The technical challenges of PD presented a barrier to the widespread adoption of an MI approach.

The limitations of conventional laparoscopy, its two-dimensional vision, restricted instrument articulation, and ergonomic challenges, have raised concerns about increased morbidity and mortality when performed outside of specialized teams. The introduction of robotic-assisted surgery, with its three-dimensional high-definition visualization, wristed instruments that mimic the human hand’s dexterity, and improved ergonomics, was heralded as a potential solution [12,13]. It was hypothesized that the robotic platform could overcome the technical hurdles of LPD, making a complex MI resection more accessible, reproducible, and safe [14]. Consequently, robotic pancreatoduodenectomy (RPD) began to gain traction, promising to accelerate the transition from open to MI pancreatic surgery [15].

For years, the literature on MIPD was dominated by retrospective cohort studies and case-matched series from high-volume institutions. While these studies often suggested benefits such as reduced intraoperative blood loss (IBL) and shorter LOS for LPD and RPD compared to OPD, they were inherently limited by selection bias, surgeon expertise, and institutional volume [14,16,17,18,19,20]. The critical question remained unanswered: were the observed improvements due to the MI approach itself, or simply a reflection of the fact that these procedures were being performed by super-specialized surgeons on highly selected patients?

The arrival of the first randomized controlled trials (RCTs) marked a critical turning point, providing the first high-level evidence to inform this debate. Initial trials yielded conflicting results. Some, like the LEOPARD-2 trial, raised significant safety concerns [21], while others from high-volume centers in Asia demonstrated feasibility and some benefits [22]. This evolving and sometimes contradictory evidence base creates a pressing need for a comprehensive synthesis.

Our analysis will be framed around several central questions that together define the strength and limits of the current evidence.

First, we will examine safety, asking whether LPD and RPD compromise patient outcomes, particularly mortality and major complications, when directly compared with OPD in randomized settings.

We will then consider efficacy, focusing on the tangible benefits in recovery and perioperative performance, including LOS, IBL, and operative time.

Equally important are the trade-offs, meaning the costs associated with these potential advantages, measured through conversion rates and financial implications.

We will also address oncological integrity, evaluating whether minimally invasive pancreatic surgery can reliably uphold the essential oncologic principles of achieving an R0 resection and performing an adequate lymphadenectomy.

Finally, we will explore generalizability, assessing whether the findings from these RCTs can be meaningfully extended to the broader surgical community or whether their applicability remains confined to ultra-specialized, high-volume centers.

By navigating the landscape of level-I evidence, this review seeks to provide a clear, evidence-based narrative on the journey from open to robot-assisted pancreatoduodenectomy, delineating the proven benefits, acknowledging the persistent challenges, and identifying the future directions for this dynamic field.

## 2. Literature Search and Selection Methodology

As a narrative review, this article aims to provide a comprehensive and critical synthesis of randomized controlled trials (RCTs) comparing open (OPD), laparoscopic (LPD), and robotic pancreatoduodenectomy (RPD). Study identification was guided by a structured but non-systematic literature exploration of major electronic databases, including PubMed/MEDLINE, the Cochrane Library, and Scopus, with the aim of identifying the major RCTs directly addressing these surgical approaches. Search terms included combinations of “pancreaticoduodenectomy,” “Whipple procedure,” “laparoscopic,” “robotic,” and “randomized controlled trial.” Study selection was based on predefined relevance criteria, focusing on trials that reported clinically meaningful outcomes related to safety, perioperative efficacy, oncological adequacy, and costs (Table 1). Priority was given to multicenter and landmark RCTs that have substantially informed current clinical practice.

This approach was intended to contextualize and critically interpret the available level I evidence rather than to provide a fully reproducible systematic review or quantitative meta-analysis.

## 3. The Safety Paradigm: Mortality and Morbidity in MIPD

The foremost concern in the evolution of any complex surgical technique is patient safety. For MIPD, the question of whether it introduces unacceptable risks compared to the proven safety profile of OPD has been central to the debate. Early RCTs provided conflicting answers, creating a narrative that has only recently begun to consolidate with the publication of the largest trials.

The initial landscape of MIPD was significantly shaped by the LEOPARD-2 trial, a multicenter, patient-blinded, randomized controlled phase 2/3 trial [21]. The trial was terminated early after an interim analysis revealed a concerning safety signal. The data showed a 90-day mortality rate of 10% (5/50) in the LPD group versus 2% (1/49) in the OPD group. Furthermore, major complications (Clavien–Dindo ≥ III) were more frequent in the LPD group (50% vs. 39%). This trial highlighted the importance of the learning curve and suggested that outside of ultra-specialized settings, LPD could pose a significant risk to patient safety.

In contrast to LEOPARD-2, subsequent larger multicenter RCTs have largely painted a different picture, demonstrating that in the hands of highly experienced surgeons, LPD can achieve safety outcomes equivalent to OPD. In 2021, Wang et al. published a Chinese multicenter RCT which randomized 656 patients and found no significant difference in 90-day mortality (2% in both LPD and OPD arms). The rate of major complications was also comparable (29% LPD vs. 23% OPD) [22].

Additionally, in 2024 Yoon et al. published a Korean multicenter RCT which reported comparable rates of severe complications between the LPD and OPD [26].

Data for RPD, while more limited, suggests broadly equivalent safety outcomes, though a note of caution is warranted. The EUROPA trial reported a 90-day mortality of 4.8% overall, with no significant difference between RPD and OPD [28] as reported by the RCT by Liu et al., which found a lower and equivalent 90-day mortality rate of 1% in both the RPD and OPD arms [30].

A detailed analysis of procedure-specific complications is crucial to fully appreciate the safety profile of minimally invasive techniques. A recent systematic review and meta-analysis of RCTs provides a detailed picture [31]. For the comparison between LPD and OPD, the evidence confirms comparable rates in major complications. Definitions were largely homogeneous across included RCTs: DGE was consistently defined using ISGPS 2007 criteria [4], while POPF was defined according to the ISGPS 2016 update in all modern trials [3], except Palanivelu et al. (ISGPS 2005) [32]. Therefore, comparisons across trials take into account the endpoint definitions adopted by each study.

The rates of DGE and PPH were not significantly different between the two approaches, while reported rates of clinically relevant POPF were lower in pooled analyses favoring LPD [31]. This data suggests that the technical execution of the critical anastomotic phases, particularly the challenging pancreaticojejunostomy (PJ), and the overall management of postoperative morbidity can be achieved with proficiency in LPD within the controlled settings of high-volume RCTs.

The picture for RPD, however, appears more complex and less consolidated. While some trials report comparable outcomes, the EUROPA trial identified a concerning trend, noting significantly higher rates of both POPF and DGE in the RPD group compared to OPD [28]. This divergence highlights that the theoretical advantages of the robotic platform do not automatically translate into superior clinical outcomes and may even introduce unique challenges. These findings underscore that the learning curve and specific technical factors associated with the robotic approach warrant particularly careful evaluation during its implementation.

Indeed, the apparent contradiction between the LEOPARD-2 trial and EUROPA trial with later studies is best explained by the learning curve phenomenon [21,28]. The LEOPARD-2 trial involved surgeons who had performed a minimum of 20 LPDs, a number now considered by many to be insufficient to overcome the learning curve for this procedure. In contrast, the large Chinese and Korean trials were conducted in centers with vast pre-trial experience, often exceeding 100 or even 200 procedures [26,29]. This underscores a critical point: the safety of MIPD is not inherent to the technique itself but is inextricably linked to surgeon proficiency and institutional volume.

Beyond safety, the purported advantages of minimally invasive surgery have historically centered on enhanced recovery and reduced surgical trauma. The analysis of RCTs allows for a rigorous, quantitative assessment of these benefits in the context of PD, moving beyond anecdotal claims to measurable outcomes.

## 4. The Efficacy Question: What Tangible Benefits Does MIPD Offer?

The most consistent and tangible benefit associated with MIPD is a reduction in LOS. This metric serves as a composite surrogate for faster recovery, less pain, and earlier return to baseline function.

The evidence for LPD is compelling. The large multicenter trial by Wang et al. demonstrated a statistically significant shorter median hospital stay for LPD (15 days) compared to OPD (16 days) [22]. This finding was supported by other RCTs. In 2018, Poves et al. reported a more substantial reduction (13.5 vs. 17 days) [24], and Yoon et al. found a significantly shorter time to functional recovery (TFR) (7.7 vs. 9.0 days) [26]. Additionally, a recent systematic review consolidates this evidence, calculating a pooled mean difference of −1.84 days (95% CI: −3.68 to −0.00) favouring LPD over OPD [31].

The data for RPD regarding LOS is less uniform but promising. The trial by Liu et al. showed a significant 2.5-day reduction in median LOS with RPD (11 vs. 13.5 days) [30]. However, the EUROPA trial did not find a significant difference in LOS [28]. Consequently, the pooled analysis by Valle et al. reflected this inconsistency, showing no significant mean difference in LOS for RPD compared to OPD (MD: 0.46 days) [31]. This discrepancy may be attributed to differing country-specific discharge policies. Indeed, while LOS is easily quantifiable, its reliability as a sole indicator of recovery is increasingly questioned. Consequently, there is a growing consensus that TFR is a more robust metric. TFR measures the number of days required for a patient to achieve predefined, functional milestones, such as adequate oral intake, independent mobility, and freedom from intravenous analgesia, irrespective of the actual discharge date. This provides a more objective assessment of the actual physiological benefit of the minimally invasive approach, less contaminated by administrative practices.

A key theoretical advantage of minimally invasive surgery is more precise dissection leading to reduced intraoperative hemorrhage. This was one of the most robustly confirmed benefits across MIPD techniques. The significant reduction in IBL was a key finding in some of the earliest RCTs, such as the pioneering work of Palanivelu et al. in India [23]. This trend was consistently confirmed across different RCTs [22,24].

For RPD, while the EUROPA trial did not show a significant difference [28], the study by Liu et al. reported a trend toward reduced IBL [30].

The primary trade-off for the benefits in LOS and IBL is a substantial increase in operative time (OT), a finding so consistent it becomes a defining characteristic of MIPD in its current evolution.

## 5. The Inherent Trade-Offs: Conversion Rates and Financial Costs

While the benefits of MIPD in recovery and reduced IBL are clear, a complete evaluation must account for the inherent trade-offs. These are primarily measured through conversion rates to open surgery and the significant financial costs associated with the minimally invasive approaches, particularly the robotic platform.

Conversion to an open procedure is a critical intraoperative outcome, serving as a direct indicator of the technical difficulty, patient selection challenges, and the surgeon’s position on the learning curve. Conversion rates in RCTs reveal a significant technical hurdle. Trials reported rates ranging from 2.0% in highly optimized settings to 23.5% in PADULAP trial [24]. The large Wang et al. trial also experienced a notable number of crossovers [22]. This wide range underscores that the risk of conversion is highly dependent on surgeon experience, patient anatomy (e.g., visceral obesity, aberrant vasculature), and tumor characteristics. It remains a tangible risk that must be discussed during preoperative consent.

The robotic platform was developed, in part, to overcome the technical limitations of laparoscopy. However, RCT data indicates that it does not eliminate the risk of conversion. The EUROPA trial reported a conversion rate of approximately 20.7% [28], while the trial by Liu et al. reported a lower rate of 3.7% [30]. This stark contrast again highlights the profound impact of the learning curve and institutional experience. It suggests that while the robot may offer technical advantages, achieving proficiency is demanding, and conversion remains a relevant outcome, especially during a center’s early experience.

However, despite conversion being commonly reported as an adverse outcome, it must not be considered a failure since patient safety is the primary outcome and must always be achieved, regardless of the surgical approach.

Across surgical oncology, robotic approaches are almost universally associated with longer operative times and higher upfront costs compared with laparoscopic techniques, and their economic justification largely relies on indirect savings from reduced postoperative morbidity and length of stay rather than formal cost superiority [33]. The financial implications of surgical innovation are a critical consideration in value-based healthcare. Across RCTs, cost reporting was heterogeneous: most trials reported perioperative resource utilization (operative time, length of stay, transfusions, and complications) as cost surrogates rather than performing formal cost accounting. The cost analysis differentiates sharply between laparoscopic and robotic approaches. The financial footprint of LPD is primarily driven by the prolonged operative time (increasing costs for operating room occupancy and personnel) and the use of disposable laparoscopic instruments (e.g., staplers, energy devices). While these costs are higher than those for OPD, they are often offset by the significant reduction in LOS and potential savings from reduced transfusion requirements. In high-volume centers with efficient workflows, LPD can approach cost-neutrality or even demonstrate cost savings over OPD when considering the entire episode of care.

The financial burden of RPD is substantially greater. The costs are multifaceted, including the initial purchase price of the robotic system, which runs into millions of dollars, significant ongoing costs for service contracts and software updates and robotic instruments that have a limited number of uses per patient, often exceeding the cost of their laparoscopic counterparts.

The EUROPA trial directly addressed this, explicitly reporting that RPD was associated with significantly higher costs compared to OPD [28]. While some high-volume centers argue that optimized robotic programs can lead to efficiencies that narrow the cost gap (e.g., through reduced conversion rates and shorter LOS as seen in Liu et al.), this should be interpreted in light of the fact that only selected RCTs performed formal cost analyses including robot-specific capital and maintenance components. At present, randomized evidence supports higher costs for RPD, whereas evidence that these costs are offset by superior patient-important outcomes or long-term benefits remains limited.

## 6. Oncological Integrity: Resection Margins and Lymphadenectomy in MIPD

In oncologic surgery, the primary goal is a complete oncological resection. In PD, this is measured by the achievement of a margin-negative (R0) resection and an adequate lymph node harvest for accurate staging. The transition to MI techniques raised valid concerns about the ability to perform an extensive dissection and complete oncological resection.

The R0 resection rate is one critical metric for evaluating the oncological radicality of the procedure. The collective evidence from RCTs demonstrates that LPD is not inferior to OPD in achieving clear margins. Early trials, such as Palanivelu et al. [23] reported no difference in R0 rates. This finding has been consistently reinforced by all subsequent major RCTs. The oncological-focused trial by Wang et al., which exclusively enrolled patients with pancreatic ductal adenocarcinoma (PDAC), confirmed equivalent R0 resection rates between LPD and OPD [25]. This could be attributed to the magnified, high-definition view afforded by laparoscopy, potentially allowing for more precise dissection along critical vascular planes.

The data for RPD, despite being from fewer studies, points towards oncological safety. The published trials reported comparable R0 rates between RPD and OPD [28,30].

A thorough lymphadenectomy is essential for accurate staging and prognostication. The number of retrieved lymph nodes serves as a proxy for the completeness of the dissection.

On this parameter, the evidence is remarkably consistent. Across all included RCTs comparing both LPD and RPD to OPD, no significant difference has been found in the total lymph node yield. This strongly indicates that both minimally invasive approaches can replicate the extensiveness of the nodal dissection performed in open surgery. The magnified view of minimally invasive surgery may even facilitate a more meticulous dissection in key nodal stations, such as those around the superior mesenteric artery.

While perioperative oncological metrics are reassuring, the ultimate measure of oncological efficacy is long-term survival. Currently, mature survival data from RCTs is still emerging.

The Qin et al. (2024) [27] study, reporting the 3-year follow-up of a previous RCT cohort, demonstrated the long-term oncological non-inferiority of LPD compared to OPD. Furthermore, this study suggests that the recovery advantages of LPD may facilitate an earlier initiation of adjuvant chemotherapy [27]. For RPD, long-term survival data from RCTs are not yet available. The ongoing follow-up of existing trials will be critical to provide a definitive answer.

## 7. Generalizability: Experts in a Controlled Setting or a New Universal Standard?

The compelling results from RCTs demonstrating the safety and efficacy of MIPD inevitably lead to a pivotal question: are these findings generalizable to the broader surgical community, or do they represent outcomes achievable only within the rarefied environment of ultra-specialized, high-volume research centers? A critical appraisal suggests that the answer is nuanced and that the external validity of these trials is inherently constrained by several key factors.

Virtually all the cited RCTs were conducted in settings that are not representative of the average hospital. The trials were performed in renowned, high-volume pancreatic surgery centers. For instance, the Chinese trials were multicenter studies involving institutions with vast experience, where surgeons were required to have performed a minimum of 104 LPDs or 40 RPDs prior to the trial [22,25,30]. Similarly, the Yoon et al. trial involved “expert surgeons” in tertiary centers [26]. This is a world apart from the reality of most hospitals, where pancreatic surgery volumes are lower and surgeon experience with MIPD is still evolving.

The dramatic contrast between the LEOPARD-2 trial, which was stopped for safety with surgeons at an earlier stage of the LPD learning curve (≥20 procedures), and the subsequent successful Asian trials underscores that the results of MIPD are profoundly experience-dependent [21]. The favourable outcomes reported in RCTs are likely the product of surgeons who have already surmounted the steepest part of the learning curve. Reported learning-curve thresholds range from a “feasibility” level of 25–40 procedures, to a “proficiency” level of 45–90 up to a “mastery” level of >45–90 in LPD and RPB, respectively [34,35]. Applying these techniques without a structured proctoring and mentoring program in lower-volume settings could lead to higher complication and conversion rates, mirroring the early concerns raised by LEOPARD-2.

Furthermore, the patients enrolled in RCTs represent a selected cohort, which may limit the applicability of the findings to a more complex, real-world population. RCTs employ strict inclusion and exclusion criteria to ensure patient safety and protocol uniformity. For example, LEOPARD-2 excluded suspected tumour involvement of major vasculature (portal vein, superior mesenteric vein/artery, or hepatic artery) on preoperative CT, a body mass index (BMI) > 35 kg/m^2^, and receipt of neoadjuvant radiotherapy [21]. Similarly, EUROPA excluded borderline or unresectable pancreatic head carcinoma according to NCCN criteria [28], and PADULAP excluded locally advanced tumors requiring preplanned major vascular resection [24]. In routine practice, surgeons encounter a much wider spectrum of anatomical and pathological challenges. The excellent outcomes for MIPD in RCTs may not be directly replicable in an obese patient with a bulky, inflammatory tumor encasing the mesenteric vessels. Accordingly, RCT results primarily reflect what is achievable in carefully selected candidates treated within high-volume trial settings. The results reflect what is achievable in ideal candidates operated on by ideal surgeons. The generalizability to “non-ideal” but commonly encountered scenarios, such as the frail elderly, the morbidly obese, or patients requiring vascular resection, remains largely unproven by level-I evidence.

Finally, the financial and infrastructural requirements for MIPD, especially RPD, create a significant barrier to widespread adoption. The conclusion that RPD does not increase operative time or compromise oncology is predicated on access to a multi-million-dollar robotic platform and the ability to absorb the high costs of consumables. This is unlikely to be achievable for many hospitals worldwide, particularly in public healthcare systems or low-resource settings. The financial findings of the EUROPA trial confirm that RPD is more expensive, limiting its generalizability to well-funded, specialized centers.

## 8. Unresolved Questions for Future RCTs

Despite the availability of randomized evidence, several clinically relevant questions regarding MIPD remain unanswered:

- Applicability to complex disease: the role of MI in borderline resectable tumors and in cases requiring planned vascular resection remains insufficiently addressed by RCTs.
- Patient selection beyond ideal candidates: outcomes in high-risk populations, including severe obesity, frailty, and significant comorbidity, are underrepresented in randomized evidence.
- Long-term oncologic outcomes: robust data on disease-free and overall survival beyond short- and mid-term follow-up are still limited, particularly for RPD.
- Learning curve and implementation: the optimal thresholds for safe dissemination outside expert centers, and how learning-curve effects interact with trial outcomes, remain unclear.
- Cost-effectiveness across health systems: whether higher upfront costs of robotic platforms are offset by downstream benefits across different healthcare settings requires dedicated economic analyses.
- Standardization of reporting: greater consistency in endpoint definitions, outcome reporting, and cost domains would facilitate cross-trial comparison and meta-analysis.

## 9. Conclusions

The evolution from OPD to MIPD, culminating in the robotic approach, represents one of the most significant advancements in modern pancreatic surgery. The body of evidence from RCTs now provides a framework for evaluating this transition.

In terms of safety, both LPD and RPD approaches have demonstrated that, despite a potential initial worsening of postoperative outcomes, they do not compromise patient outcomes regarding mortality and major complications when performed by surgeons who have surmounted the substantial learning curve in high-volume, specialized centers. The early safety signals served as a vital corrective, underscoring that proficiency is non-negotiable.

The efficacy of MI techniques is confirmed by tangible benefits: a consistent reduction in IBL and a shorter LOS, particularly for LPD. The robotic platform shows potential in mitigating the prolonged operative time associated with LPD. Furthermore, concerns regarding oncological integrity have been robustly addressed. Both LPD and RPD achieve R0 resection rates and lymph node yields equivalent to those of open surgery.

However, these advantages are not without their trade-offs. The technical challenge of MIPD is reflected in persistent conversion rates, and the financial expenditure, especially for RPD, remains significantly higher. Consequently, the generalizability of these excellent RCT outcomes is conditional, largely confined to expert centers with structured training programs.

In summary, the journey from OPD to RPD is not a linear progression of superior technique but an expansion of surgical options. The decision for a specific approach must be individualized, balancing patient-centric recovery benefits against the realities of technical complexity, cost, and institutional expertise. The future of MIPD lies not in replacing one standard with another, but in the thoughtful integration of these advanced techniques into specialized care pathways, ensuring that innovation consistently translates into safe and effective patient care.

## Figures and Tables

**Table 1:** Summary of randomized controlled trials comparing open vs. laparoscopic and robotic pancreatoduodenectomy.

Study	Country	Comparison	Sample Size	Primary Endpoint	Main Findings
Palanivelu et al., 2017 [23]	India	LPD vs. OPD	66	Morbidity	No significant difference in overall complications
PADULAP–Poves et al., 2018 [24]	Spain	LPD vs. OPD	64	Postoperative complications	Similar morbidity; LPD associated with shorter LOS but high conversion rate (23.5%)
LEOPARD-2–van Hilst et al., 2019 [21]	Europe	LPD vs. OPD	99	Severe complications	Higher 90-day mortality in LPD (10% vs. 2%); trial stopped early
Wang et al., 2021 [22]	China	LPD vs. OPD	656	Severe complications	LPD non-inferior to OPD; similar mortality and morbidity
Wang et al., 2023 [25]	China	LPD vs. OPD	261	R0 resection & lymph nodes	No significant differences between groups
Yoon et al., 2024 [26]	Korea	LPD vs. OPD	160	Time to functional recovery (TFR)	TFR significantly shorter after LPD
Qin et al., 2024 [27]	China	LPD vs. OPD	656	3-year survival	No differences in long-term survival
EUROPA–Klotz et al., 2024 [28]	Europe	RPD vs. OPD	80	Severe complications	Higher POPF and DGE in RPD; markedly higher costs
Liu et al., 2023 [29]	China	RPD vs. OPD	378	Length of stay	RPD associated with ~2.5-day reduction in LOS

Abbreviations: LPD = laparoscopic pancreatoduodenectomy; RPD = robotic pancreatoduodenectomy; OPD = open pancreatoduodenectomy; LOS = length of hospital stay; TFR = time to functional recovery; POPF = postoperative pancreatic fistula; DGE = delayed gastric emptying.

## Data Availability

No new data were created or analyzed in this study. Data sharing is not applicable to this article.

## Abbreviations

The following abbreviations are used in this manuscript:

DGE	Delayed Gastric Emptying
IBL	Intraoperative Blood Loss
ISGPS	International Study Group of Pancreatic Surgery
LPD	Laparoscopic Pancreatoduodenectomy
LOS	Length of (Hospital) Stay
MI	Minimally Invasive
MIPD	Minimally Invasive Pancreatoduodenectomy
OPD	Open Pancreatoduodenectomy
OT	Operative Time
PD	Pancreatoduodenectomy
PDAC	Pancreatic Ductal Adenocarcinoma
PJ	Pancreaticojejunostomy
POPF	Postoperative Pancreatic Fistula
PPH	Postpancreatectomy Hemorrhage
R0	Microscopically margin-negative resection
RCT	Randomized Controlled Trial
RPD	Robotic Pancreatoduodenectomy
TFR	Time to Functional Recovery
CD	Clavien–Dindo (classification)
